# Advances in the study of the interaction between schistosome infections and the host's intestinal microorganisms

**DOI:** 10.1186/s13071-024-06245-1

**Published:** 2024-04-10

**Authors:** Ao Hong, Abdulrahim Umar, Hao Chen, Zheng Yu, Jing Huang

**Affiliations:** 1 Department of Parasitology, School of Basic Medical Science, Central South University, Changsha, China; 2 Human Microbiome and Health Group, Department of Microbiology, School of Basic Medical Science, Central South University, Changsha, Hunan China; 3 China-Africa Research Center of Infectious Diseases, Central South University, Changsha, Hunan China

**Keywords:** Schistosomiasis, Intestinal microbiome, Schistosome–gut microbiome interactions, Gut–liver axis, Probiotic

## Abstract

Schistosomiasis, also called bilharziasis, is a neglected tropical disease induced by schistosomes that infects hundreds of millions of people worldwide. In the life cycle of schistosomiasis, eggs are regarded as the main pathogenic factor, causing granuloma formation in the tissues and organs of hosts, which can cause severe gastrointestinal and liver granulomatous immune responses and irreversible fibrosis. Increasing evidence suggests that the gut microbiome influences the progression of schistosomiasis and plays a central role in liver disease via the gut–liver axis. When used as pharmaceutical supplements or adjunctive therapy, probiotics have shown promising results in preventing, mitigating, and even treating schistosomiasis. This review elucidates the potential mechanisms of this three-way parasite–host–microbiome interaction by summarizing schistosome-mediated intestinal flora disorders, local immune changes, and host metabolic changes, and elaborates the important role of the gut microbiome in liver disease after schistosome infection through the gut–liver axis. Understanding the mechanisms behind this interaction may aid in the discovery of probiotics as novel therapeutic targets and sustainable control strategies for schistosomiasis.

**Graphical Abstract:**

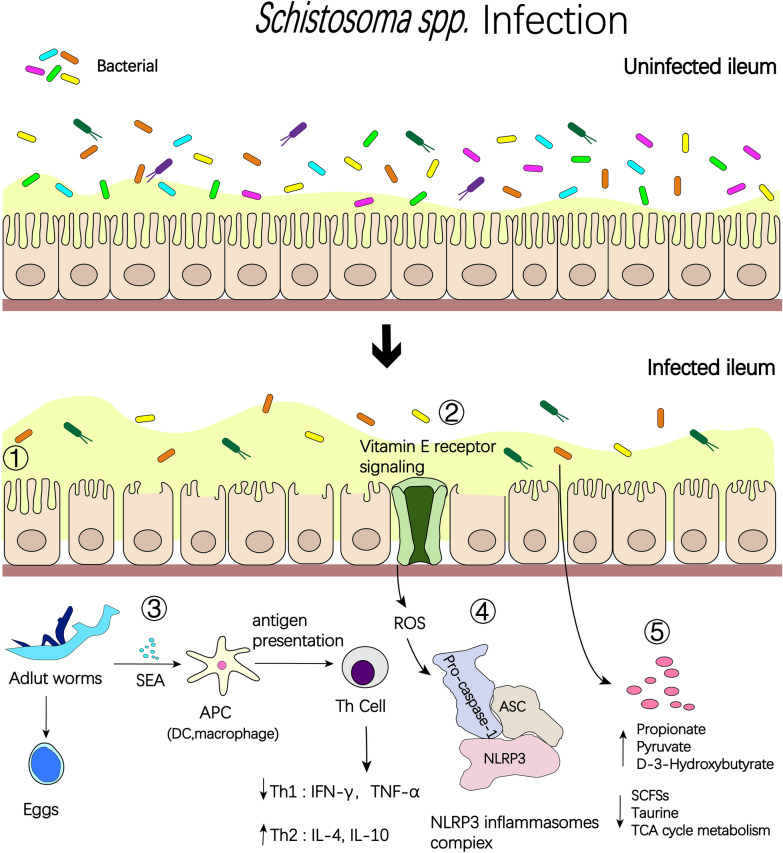

## Background

Schistosomiasis is a globally neglected tropical disease (NTD) that affects both humans and animals, and is prevalent in tropical and subtropical regions [1]. According to estimates from the World Health Organization (WHO), at least 251.4 million people required preventive treatment in 2021 to combat this disease [2–4]. The main schistosomes infecting humans are *Schistosoma mansoni*, which is transmitted by *Biomphalaria* snails and causes intestinal and hepatic schistosomiasis in Africa, the Arabian peninsula, and South America; *Schistosoma haematobium*, transmitted by *Bulinus* snails and causing urinary schistosomiasis in Africa and the Arabian peninsula; and *Schistosoma japonicum*, transmitted by the amphibian snail *Oncomelania* and causing intestinal and hepatosplenic schistosomiasis in China, the Philippines, and Indonesia. The life cycle of schistosomes involves a human or other mammal as a final host and a freshwater snail as intermediate host [1]. The human population is infected with schistosomes through contact with contaminated water, including cercariae. Schistosome infections can result in host damage caused by various stages of the parasite's life cycle, including cercariae (larval stage), larvae, adult worms, and eggs. The secretions and excretions of each of these stages can be used as antigenic substances to induce a series of humoral and cellular immune responses that can cause complications of infection [5]. Adult worms of *S. japonicum* and *S. mansoni* inhabit the portal-mesenteric venous system, while adult worms of *S. haematobium* primarily parasitize the bladder plexus. Eggs are the main pathogenic factors of schistosomiasis, and *S. japonicum* and *S. mansoni* eggs are deposited mainly in the intestinal wall and liver tissue of the host, causing gastrointestinal and hepatosplenic diseases, while *S. haematobium* eggs are deposited mainly in the bladder wall and genital organs of the host, causing urogenital diseases [3].

Intestinal schistosomiasis is one of the complications caused by schistosome infections. The adult worms of *S. mansoni* and *S. japonicum* live in the mesenteric veins, and their mating and egg-laying behavior can cause intestinal schistosomiasis. Symptoms of intestinal schistosomiasis are related to the migration or deposition of eggs through or in the intestinal tissues. Eggs retained in the intestinal wall secrete a series of antigenic mixtures that promote the convergence of immune cells and the infiltration of inflammatory factors, which results in the formation of granulomas in the tissues around the site of egg deposition, accompanied by ulceration and superficial hemorrhage [6]. This disease is characterized by symptoms such as abdominal pain, loss of appetite, and diarrhea [3], and if left untreated, it can lead to extensive fibrosis and even liver and spleen disease. The mammalian gut harbors a diverse community of microbiota that play a crucial role in regulating host immunity through their surface antigens or small-molecule metabolites [7]. Accumulating evidence indicates that many gut microorganisms may influence multiple aspects of host physiology, such as metabolism and immunity [8, 9]. These resident microorganisms maintain homeostasis within arthropod vectors via a variety of immune mechanisms while playing intricate roles in the regulation of host susceptibility to schistosomes in direct and/or indirect ways [9]. The intestinal tract of mammals is a pivotal site for the life cycle of schistosomes, in which metabolites of the intestinal flora can modulate the host immune response and thereby reduce the severity of schistosomiasis and its complications [10]. Such intricate interactions among hosts, their symbiotic microbiota, and schistosomes have recently garnered considerable attention, as the microbiota could be exploited for disease transmission control.

At present, there is no targeted vaccine available in clinic, and the control strategy for schistosomiasis relies primarily on the administration of a single anthelmintic drug, praziquantel (PZQ), in a mass dosing program [11]. At the WHO-recommended dose of 40 mg/kg, the cure rate (CR) for PZQ was reported at 73.6% for *S. haematobium*, 76.4% for *S. mansoni*, and 94.7% for *S. japonicum* [12]. However, while PZQ is effective in treating the disease, it exhibits low killing efficacy on juvenile worms, and the potential emergence of its drug resistance is a continual concern [13]. Therefore, new and sustainable strategies are needed to control schistosomiasis. While worm eggs and host immune response are the two most important factors driving granuloma formation, recent studies have identified gut microbiota as a third factor. Recent evidence suggests that the host gut microbiota plays a crucial role in the interaction between schistosomes and host immunity [10]. Schistosome infections lead to granuloma and fibrotic lesions in the intestine, which is also the site of survival for the intestinal microbiota [3]. The relationship between schistosome infections and host intestinal microbiota is a vigorously investigated area of research, and it has the potential to provide new insights into the course of infection of the disease.

In this paper, we review the current research on schistosome infections and host gut–microbial interactions, and recent progress in studies on the role of the gut microbiota in the regulation of schistosome infections. Understanding these sophisticated interactions may offer new strategies for the prevention of this neglected tropical disease.

### Schistosome infections disrupt the host intestinal microorganisms

**Fig. 1:**
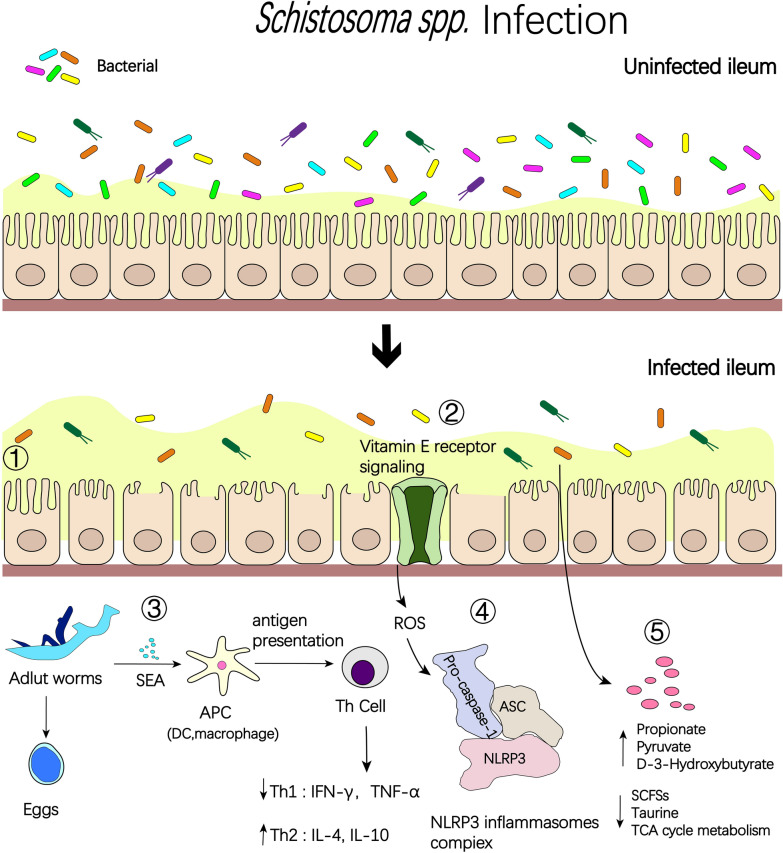
During schistosome infection, inflammatory processes play an important role in parasite–microbiota interactions and their consequences. (1) Degradation of barrier function by the schistosome allows bacterial translocation and consequent inflammation, while microbe-driven pro-inflammatory responses contribute to antiparasitic immunity. (2) Decrease in bacterial abundance and diversity. (3) After the eggs mature in the worm’s body, their constantly secreted enzymes, proteins, and sugars and other soluble egg antigens penetrate the surrounding tissues through the microscopic pores of the eggshell, are phagocytosed and processed by macrophages and presented to T helper (Th) cells, while secreting interleukin-1 (IL-1), which activates Th and causes it to produce a variety of cytokines, and as the schistosome infection progresses, cytokine cascades change from a Th1 to Th2 profile. (4) Vitamin E may alleviate inflammatory damage by inhibiting the production of reactive oxygen species and generating NOD-like receptor thermal protein domain associated protein 3 (NLRP3) immune complexes. (5) Metabolic changes surrounding schistosome infection

Understanding the host's gut microbial composition and the factors that influence it is crucial to unraveling the intricate relationship between the human body and its resident microorganisms. The intestinal flora of mammals is dominated by Firmicutes, Bacteroidetes, and Proteobacteria, with *Lactobacillus* having the highest abundance among Firmicutes. Intestinal flora structure also varies with season, sex, reproductive status, body mass index, host infection status, and anatomical site of the intestine [14, 15]. When infected with schistosomes, the diversity of the host's intestinal flora reflects the host's health status, with a higher diversity index indicating better host metabolism and immune system function, whereas a decrease in the diversity of the intestinal flora leads to intestinal dysbiosis and a decrease in both metabolic and immune function, which can lead to more serious complications [16]. Changes in host intestinal flora diversity can be observed during schistosome infections of the host (Fig. 1).

Specifically, it was observed that *S. japonicum* parasitizes human and mammalian venous vessels and also significantly reduces intestinal microbial alpha diversity in C57BL/6 mice infected with *S. japonicum*. On the other hand, alpha diversity increases in acutely infected BALB/c mice [8]. Additionally, intestinal flora beta diversity is increased in different mouse strains during different infection periods. Intestinal granulomas also play a role in shaping the microbial communities: the abundance of Firmicutes decreases, the abundance of *Bacteroides* increases during acute infection but decreases during chronic infection, and the abundance of *Lactobacillus* gradually decreases during the infection period [8]. These findings align with the results reported by Zhang et al. [9] which indicated a reduction in the diversity of the intestinal microbiota in mice infected with *S. japonicum* when compared to normal, uninfected mice. Taxonomic analysis showed that the abundance of Firmicutes decreased with infection, while the abundance of Bacteroidetes increased with infection [17]. In contrast, after infection with *S. japonicum*, the relative abundance of Proteobacteria increased with increasing severity of infection. It has been well documented that the relative abundance of Proteobacteria in the intestinal flora also increases in other disease states, so researchers suggest that an increased abundance of Proteobacteria could be a diagnostic marker for intestinal flora imbalance and potential diseases [18].

Studies have shown that during the invasive phase of *S. mansoni* infection, close contact between adult worms, eggs, or larvae and the intestinal mucosa of the host can trigger a strong local inflammatory response in the organism, leading to changes in intestinal flora homeostasis, with a decrease in microbial alpha diversity, an increase in Bacteroidetes, and a decrease in *Lactobacillus* [19]. In mice infected with a mixture of *S. mansoni* females and males, intestinal microbial alpha diversity was reduced and beta diversity was significantly increased, with increased abundance of *Akkermansia muciniphila*, *Lactobacillus*, *Alistipes*, and *Bacteroides* [20, 21]. The intestinal flora of mice with mono-infection of male worms, although also affected, was less severe than in mixed infections, with a community structure more like that of normal mice.

In 2021, Ajibola et al. reported that infections caused by *S. haematobium* decreased the abundance of Firmicutes and increased the abundance of Proteobacteria—a shift in community structure associated with ecological dysregulation similar to the other two schistosome infections. Specifically, the authors detected many changes similar to inflammation-related ecological dysregulation in lower taxa, including decreases in *Clostridiales* and increases in *Moraxellaceae*, *Veillonellaceae*, *Pasteurellaceae*, and *Desulfovibrionaceae* [22]. Similarly, a study found that genitourinary schistosomiasis caused by *S. haematobium* was associated with changes in the abundance of several gut microorganisms in preschool children (1–5 years) in Zimbabwe. 16S rRNA sequencing showed that *Prevotella* and *Aspergillus* were more abundant in children infected with *S. haematobium* than in uninfected children. More specifically, the fecal abundance of *Pseudomonas*, *Stenotrophomonas*, and *Aspergillus* increased, and the abundance of *Azospirillum* decreased [23]. Summarizing previous studies, it has been found that the infection and life history of different strains of *Schistosoma* can affect the host's intestinal flora.

We summarize the intestinal commensal bacteria and their association with schistosomiasis in Table 1. It has been well documented that Firmicutes and Proteobacteria are the most responsive phyla in schistosomiasis [18]. Additionally, it has been established that the relative abundance of Proteobacteria in intestinal flora increases during other disease states. Researchers believe that this increase can be used as a diagnostic marker for intestinal flora imbalances and potential diseases [24, 25]. Specifically, *Methylophilus* is considered an aerobic, methanol-using bacterium and one of the most important sugar-decomposing species, expressing several antioxidant enzymes [26]. These findings suggest that the abundance of *Methylophilus* in schistosomiasis patients may be a consequence of the inflammatory response to acute schistosomiasis, and its increase could serve as a marker for dysbiosis of intestinal flora following schistosomiasis infection. Furthermore, the increased relative abundance of *Turicibacter* in schistosomiasis-infected patients suggests its involvement in the development of systemic inflammation through altered immune cell activation [27]. However, the relative abundance of *Clostridium*, *Butyricimonas*, and *Veillonella* was reduced in infected patients. A previous study suggests that reduced levels of *Clostridium* are associated with total cholesterol levels [28], suggesting these taxa may play a role in the infection by affecting lipid metabolism. Additionally, the relative abundance of *Butyricimonas* and *Veillonella* has been negatively correlated with the severity of many diseases [29]. There is also a unique phylum, Saccharibacteria (formerly TM7), that is globally distributed and is commonly associated with inflammatory mucosal diseases in humans, and the TM7 phylotype is able to inhibit the production of tumor necrosis factor alpha (TNF-α), suggesting its potential immunosuppressive capacity [30]. Previously unrelated to schistosome infections, it was found to be increased in relative abundance in patients with acute *S. japonicum* infection [18], suggesting that increased relative abundance of TM7 may be a novel biomarker associated with *S. japonicum* infection. Therefore, changes in the relative abundance of these taxa suggest that the microbiota are associated with changes in metabolic and immune responses during schistosome infections.

**Table 1:** Intestinal commensal bacteria and their association with schistosomiasis

Phylum	Family/genus/species	Host	Observations	References
Proteobacteria	*Comamonas* and *Psychrobacter*	Adults	Negatively correlated with parasite burden	[18]
	*Lachnospiraceae_NK4A136*	BALB/c mice	Negatively correlated with parasite burden	[17]
	*Ruminiclostridium*	BALB/c mice	Negatively correlated with parasite burden	[17]
	*Methylophilus*	Adults	An inflammatory response to acute schistosomiasis, positively correlated with parasite burden	[18]
	*Salmonella typhimurium* (ATCC14028)	Children	Reduced the number of adult schistosomal worms and eggs and relieved symptoms of schistosomiasis	[103]
Firmicutes	*Bacillus amyloliquefaciens*	BALB/c mice	Modulated disease severity	[86]
	*Bacillus subtilis*	BALB/c mice	Modulated disease severity	[87]
	*Butyricimonas *and* Veillonella*	Adults	Negatively correlated with parasite burden	[18]
	*Clostridium*	Adults	Correlated with total cholesterol levels, negatively correlated with parasite burden	[18]
	*Faecalibacterium*	Adults	Negatively correlated with liver damage in patients with *S. japonicum* infection	[104]
	*Lactobacillaceae*	*Microtus fortis*	A positive role in the natural resistance to schistosome infection, positively correlated with parasite burden in *Microtus fortis*	[9]
	*Megamonas*	Adults	A biomarker for diagnosis which helped to discriminate different stages of *S. japonicum* infection	[105]
	*Muribaculaceae*	*Microtus fortis*	A negative role in the natural resistance to schistosome infection, negatively correlated with parasite burden in *Microtus fortis*	[9]
	*Roseburia*	C57BL/6 mice	Positively correlated with *S. mansoni* infection burden	[66]
	*Ruminococcus*	C57BL/6 mice	Negatively correlated with infection burden	[66]
	*Shuttleworthia*	C57BL/6 mice	Negatively correlated with *S. mansoni* infection burden	[66]
	*Turicibacter*	Adults	An inflammatory response to acute schistosomiasis, positively correlated with parasite burden	[18]
Bacteroidetes	*Alistipes*	BALB/c mice	Depleted in *Salmonella*-infected mice, led to the destruction of the intestinal barrier	[31]
	*Bacteroides*	Adults	Activated an infectious response in the intestine, and they were significantly more abundant in a mouse model of *S. mansoni* infection, displayed a significant correlation with the level of hepatic granulomas	[18, 31]
	*Prevotella*	Adults and children	A biomarker for diagnosis which helped to discriminate different stages of *S. japonicum* and *S. haematobium* infection	[66, 104]
	*Sphingobacterium*	Adults	Identified as a marker in urogenital schistosomiasis	[106]
Fusobacteriota	*Fusicatenibacter*	Adults	A biomarker for diagnosis which helped to discriminate different stages of *S. japonicum* infection	[105]
*Veillonellaceae*	*Akkermansia muciniphila*	C57BL/6 and BALB/c mice	Positively associated with *S. mansoni* infection	[8, 20]
Saccharibacteria (TM7)	Unclassified	Adults	A novel biomarker associated with *S. japonicum* infection	[18]
Unclassified	*Subdoligranulum*	Adults	A biomarker for diagnosis which helped to discriminate different stages of *S. japonicum* infection	[105]

Based on results from mouse and human gut microbiomes (Table 1), the microbiota associated with fibrosis or granulomas, Bacteroidetes and *Enterococcus*, were found to correlate with the level of *S. japonicum*-induced liver injury and could be used to differentiate between *S. japonicum* infections. Interestingly, patients with acute *S. japonicum* infection had a higher proportion of a *Bacteroides*-rich enterotype than healthy controls [31]. This phenotype is associated with a wide range of glycolytic potentials, including the presence of genes encoding enzymes such as proteases, hexokinases, and galactosidases, and given these enzymatic potentials, it seems likely that these organisms derive energy from dietary carbohydrates and proteins [32]. Increasing evidence suggests that Bacteroidetes activate the intestinal infection response [33], and they were significantly more abundant in a mouse model of *S. mansoni* infection [20]. Based on the altered immune response in acute *S. japonicum* infection [34] and data on intestinal microbial regulation in mice after *S. japonicum* infections [18], studies should examine whether altered phenotypes correlate with the *Bacteroides* enterotype as a possible biomarker for acute schistosomiasis. Subsequently, *Veillonellaceae* was detected as the dominant phylum in mice, and a significant expansion of *Akkermansia muciniphila* was associated with *S. mansoni* infection [20]. Similar results were found in humans after broad-spectrum antibiotic treatment [35]. The current findings provide additional insights into the gut microbiota of various hosts following *S. japonicum* infection and demonstrate the potentially powerful role of microbiome analysis for the early and accurate diagnosis of schistosomiasis and for understanding the causative mechanisms of disease progression.

### Changes in metabonomic investigation

Most microbial metabolites are derived mainly from carbohydrate and protein fermentation. Short-chain fatty acids (SCFAs) are one of the most common microbial metabolites, derived from non-digestible carbohydrates [36]. Reduced concentrations of SCFAs such as acetate, butyrate, and propionate are a characteristic finding in urine samples obtained from *S. mansoni*-infected mice [37]. These SCFAs are produced by bacteria in the colon by fermenting unabsorbed dietary fiber, providing a source of energy for metabolism in the colon and liver, and are involved in the progression of schistosome infections. Studies have shown that both glycolipid and amino acid metabolism are affected in hosts infected with these schistosomes and that host gut microbial metabolism is disturbed. Nuclear magnetic resonance (NMR) analysis of urine samples from *S. mansoni*-infected mice revealed a decrease in the concentration of tricarboxylic acid cycle (TCA cycle) intermediates and an increase in pyruvate levels. These findings suggest that the glycolytic pathway was affected, and there were disruptions in amino acid metabolism and microbial metabolism [37]. Additionally, infected mice exhibited increased amino acid concentrations and membrane phospholipid metabolites, and decreased energy-related metabolites in their tissues [38]. Urinary metabolic profiles of *S. mansoni*-infected mice were analyzed using NMR and capillary electrophoresis, identifying 3-ureidopropionate, p-cresol glucosinolate, phenylacetylglycine, indophenol sulphate, isocitric acid, and trimethylamine (TMA) as markers for distinguishing infected mice from their healthy counterparts [39, 40]. Further NMR-based metabolomic studies of *S. mansoni*-infected populations revealed that infected and uninfected individuals could be differentiated based on variations in urinary metabolic profiles. Potential biomarkers of infection were mainly associated with altered intestinal flora, energy metabolism, and liver function [41].

The metabolic changes induced by *S. japonicum*-infected hosts were similar to those of *S. mansoni*, and the metabolic response to *S. japonicum* infection was generally consistent across suitable hosts. In golden hamsters infected with *S. japonicum*, NMR revealed a decrease in TCA cycle intermediates and an increase in pyruvate levels in the urine of hamsters, and microbial metabolism was affected, consistent with that of mice infected with *S. mansoni*; the synthesis or utilization of SCFAs was also inhibited in the former [42]. The dynamic metabolic profiles of plasma, urine, and liver of BALB/c mice infected with *S. japonicum* were analyzed using NMR assays and revealed that infection led to increased concentrations of 3-ureidopropionate (a uracil catabolic product) in urine, disturbances in lipid metabolism, activation of glycolysis, inhibition of the TCA cycle, and disturbances in the regulation of intestinal flora. The changes in 3-ureidopropionate and overall changes in metabolites in urine and plasma samples were closely related to the development of liver fibrosis [43]. The metabolic profile of BALB/c mice infected with *S. japonicum* was investigated, revealing significant alterations in amino acid metabolism, DNA and RNA synthesis, phospholipid metabolism, glucose metabolism, and intestinal microbial metabolism in the serum of infected mice. Moreover, the study identified 17 specific metabolites closely associated with the development of schistosomiasis [44]. However, in mice co-infected with *S. japonicum* and *Salmonella typhimurium*, it was observed that infection with *S. typhimurium* eliminated some of the adult worms and eggs, reduced the symptoms of schistosomiasis and mortality in mice, and ameliorated the metabolic disorders caused by schistosomiasis in mice [45].

The most important clinical complication of schistosome infections is periportal liver fibrosis, but less research has been done on how intestinal microorganisms act in the liver via the gut–liver axis after schistosome infections. In a *S. mansoni* mouse model, Wang et al. observed impaired hepatic function of urinary metabolites in mice at day 49 post-infection. Depleted levels of 2-oxo isovaleric acid and 2-oxo isodecanoic acid, derived from valine and leucine, respectively, were observed in *S. mansoni*-infected mice [46], suggesting a disturbance in amino acid metabolism. This finding is consistent with the elevated serum levels of leucine in patients with *S. haematobium* infection [47]. A decrease in D-3-hydroxybutyrate is another indication of impaired liver function, as the liver mainly produces D-3-hydroxybutyrate during fatty acid oxidation. A few studies have examined the effect of bacterial metabolites on the progression of schistosome infections. It was recently shown that the reduced abundance of some SCFA-producing bacteria correlates with the progression of schistosome infections [31]. To date, few studies have investigated the correlation between schistosome infections and changes in gut metabolomics, and additional studies are needed to elucidate the possible role of gut microbial-derived metabolites in schistosome progression. We summarized the changes in metabolites observed in urine after schistosome infections, as shown in Table 2.

**Table 2:** Changes in metabolites observed in urine

	Metabolite	Related pathway	Trend	References
Gut microflora	Dimethylamine	Carbon metabolism; methane metabolism	↑or↓^a^	[38, 41]
	Hippurate	Bile secretion	↓	[38, 41]
	Phenylacetylglycine (PAG)	Phenylalanine metabolism	↑	[37, 43]
	Trimethylamine	Carbon metabolism; methane metabolism	↑	[41, 43]
Energy metabolism	2-Oxoglutarate	Citrate cycle (tricarboxylic acid [TCA] cycle)	↓	[37, 38, 107]
	Acetate	Citrate cycle (TCA cycle)	↓	[41, 43]
	Alanine	Phenylalanine metabolism; protein digestion and absorption	↑	[37, 38]
	Citrate	Citrate cycle (TCA cycle)	↓	[104, 108]
	Fumarate	Citrate cycle (TCA cycle)	↓	[38, 104]
	Pyruvate	Citrate cycle (TCA cycle)	↑	[38, 41]
	Succinate	Citrate cycle (TCA cycle); carbon metabolism	↓	[38, 41]
	Citrate	Citrate cycle (TCA cycle); carbon metabolism	↓	[37, 43]
Liver function	Acetone	Amino acid metabolism	-	[37, 43]
	Creatine	Amino acid metabolism	↑	[41, 43]
	Methylguanidine	ND	↑	[41, 43]
	2-Oxo isocaproate	Biosynthesis of amino acids	↓	[41, 43]
	3-Hydroxy butyrate	Degradation of amino acids	↓	[41, 43]
Other	Formate	Pyruvate metabolism	↓	[41, 43]
	Trimethylamine *N*-oxide (TMAO)	Methane metabolism	↑	[41, 43]
	Trigonelline	Tropane, piperidine, and pyridine alkaloid biosynthesis	↓	[38, 41]

ND: No metabolic pathways involved in this metabolite were found in this study

^a^Dimethylamine increased after *S. japonicum* infection but decreased after *S. mansoni* infection

### *Schistosoma*–bacterial interactions

Schistosomes manipulate the innate and adaptive immune system of the host for their own benefit [48]. Similarly, bacteria employ various strategies to modulate the host immune response [49]. A study suggested that symbiotic bacteria in the host play a significant role in the formation of intestinal granulomas and the specific immune response during *S. mansoni* infection, which may impact the excretion of eggs. Cases of co-infection with intestinal bacteria and schistosomes have been reported, where intestinal bacteria, once in the bloodstream, can reach the adult schistosome worms residing in the mesenteric veins and establish colonization in the parasite's cecum [50]. The most frequently reported association is between *Schistosoma* and *Salmonella* infection, with most studies consistently finding that prior schistosome infections enhance and prolong subsequent *Salmonella* infection. In particular, co-infected hosts show higher bacteremia, increased virulence, higher mortality rates, and more persistent local liver or spleen infections than hosts infected with only *Salmonella* [51]. The association between *Salmonella* and *Schistosoma* leads to prolonged bacterial infection, development of antibiotic resistance, and poor treatment outcomes for both infections [52]. In addition, there are mixed infections involving *Helicobacter pylori*, and interestingly, different research studies have shown a “beneficial” effect of co-infection with *Schistosoma* *spp*. and *H. pylori* [53, 54]. Compared to patients with single infections of either *H. pylori* or *Schistosoma*, patients with mixed infections exhibit less severe gastritis and reduced gastric mucosal damage and changes in serum reactions related to the risk of gastric atrophy [55]. During the acute phase of schistosome infection, a decrease in the number of T cells in the stomach is observed. In hosts infected only with *H. pylori*, T cells accumulate in the stomach and cause inflammation. The study demonstrated that schistosomiasis increases the levels of chemokines in the liver, which attract T cells to aggregate in the liver and reduce gastric inflammation [55]. However, this influence gradually diminishes as the chronic phase begins. Another study detected increased levels of the signaling protein IL-13dRa2 in the blood of mice infected with *H. pylori *[56]. IL-13dRa2 can offer protection against liver fibrosis and even reverse the progression of tissue remodeling. At first glance, the interaction during co-infection may appear as a “beneficial” side effect: although the infected individual suffers from two diseases, the harmful effects of both seem to be reduced. However, co-infection may have other consequences: the complexity of immune responses can limit the effectiveness of vaccines [56].

The available information on the interaction between bacteria and *Schistosoma* is somewhat limited. However, increasing evidence suggests that this interaction deserves further attention, especially considering the growing understanding of schistosomiasis. In vitro experiments conducted in the 1980s demonstrated the natural co-infection of *Schistosoma* and *Salmonella*, with bacteria adhering to the surface membrane of *S. mansoni* [57]. However, recent studies have indicated that *S. typhimurium* infection in mice reduces the number of adult schistosome worms [58]. Barnhill and colleagues explored the potential benefits of bacterial adherence to the schistosome’s outer shell [52]. They acknowledged that simultaneous infection by *Schistosoma* and *Salmonella* in humans is common and further investigated this through in vitro experiments. Their experiments using isolated adult *S. mansoni* demonstrated that bacteria attached to the parasite's surface were not affected by antibiotic treatment. This interaction between bacteria and *Schistosoma* appears to be specific, as the presence of mammalian cells or flatworms does not provide antibiotic protection.

Another emerging area of interest is the microbial communities that reside on parasitic worms, collectively known as the worm-associated microbiota [59]. To date, only one study has demonstrated the presence of bacterial communities on the surface of adult *S. japonicum* by fluorescence in situ hybridization (FISH) and microbiome profiling. These bacteria on the surface of schistosomes are distinct from the host's blood microenvironment and should be regarded as an important new component of the interaction between the host and the parasite, although their function remains unclear [60]. Some bacterial species on the surface of schistosomes may represent additional immune evasion mechanisms. *Streptococcus*, *Haemophilus influenzae*, *Escherichia coli* K1, and *Neisseria meningitidis* all have structures that prevent antibody adhesion and complement insertion on their surfaces [61]. Among these groups, *Escherichia coli* (not K1) exists on the shell and body of the parasite. Secretions from Gram-negative bacteria may be another defense mechanism beneficial to schistosomes. The presence of multiple layers of membranes at the top of adult schistosomes can make them resistant to toxins secreted by bacteria [62], but this may also cause damage to host cells, especially those involved in immune responses. Based on this study, Formenti et al. from the University of Cambridge applied FISH to investigate the occurrence of *S. mansoni*-associated bacteria throughout key developmental stages [63]. They detected strong 16S rRNA signals associated with the tip/lateral glands of eggs and miracidia, to the acetabular glands and oral/ventral suckers of cercariae, and to the gut and tegument of adult worms [63]. This finding suggests that the reproduction of *S. mansoni*-associated bacteria may occur vertically through eggs. In addition, we suggest some mechanisms by which bacteria may kill adult *S. mansoni*, including (1) muscle damage in the parasite, (2) mechanical tissue damage caused by a large number of bacteria settling in the cerebral cortex, (3) bacterial endotoxin secretion, and (4) competition for essential nutrients and/or metabolites. The latter hypothesis implies that externally acquired bacteria may compete with schistosome-associated bacteria for space and nutrients, leading to significant disruptions in the microbiome balance of the parasite.

A study from the UK has revealed an overlooked aspect of the biology of schistosomes: adult parasites reside in the blood of mammalian hosts, playing a positive role in regulating the host's immune system and obtaining necessary nutrients [60]. The adult schistosome worms appear to alter their interaction with the blood microbiome, contributing to the interaction between the host and schistosomes after parasitism. Researchers have shown that the microbiomes on the surface of adult schistosome worms differ from those found in the host's blood microenvironment. The concentration of bacterial populations increases on the surface of schistosomes, while other bacterial groups are absent, representing a newly discovered microbial niche. Furthermore, functional differences between the microbiome of the schistosome gut epithelium and its tegument deepen the complex interplay between schistosomes, hosts, and microbial populations [64, 65].

### Understanding the gut–liver axis model for schistosomiasis

**Fig. 2:**
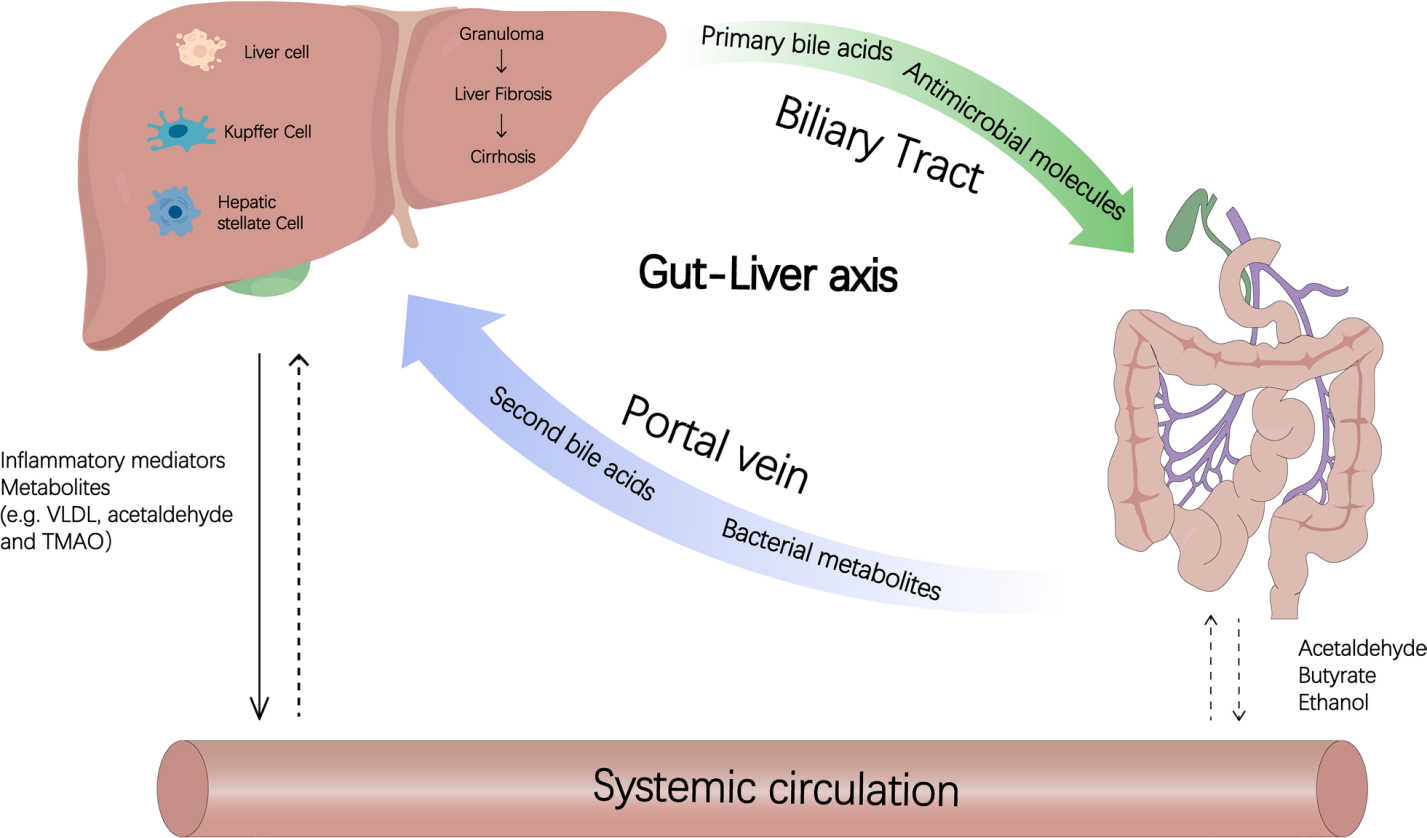
Speculation on the gut–liver axis after schistosome infections. The liver transports bile salts and antimicrobial molecules to the intestinal lumen through the biliary tract. This process maintains gut eubiosis by controlling unrestricted bacterial overgrowth. Bile salts also act as important signaling molecules via nuclear receptors to modulate hepatic bile acid synthesis, glucose metabolism, lipid metabolism, and energy utilization from diet. Conversely, gut products, such as host and/or microbial metabolites and microbial-associated molecular patterns, translocate to the liver via the portal vein and influence liver functions. Additionally, systemic circulation extends the gut–liver axis by transporting liver metabolites from dietary, endogenous, or xenobiotic substances to the intestine through the capillary system. Owing to this medium of transport and ease of diffusion of systemic mediators across blood capillaries, these factors could affect the intestinal barrier positively (for example, butyrate) or negatively (for example, acetaldehyde)

Schistosome infection induces ecological dysbiosis of the gut microbiota in animals and humans, as well as acute or chronic liver injury, such as advanced schistosomiasis, liver fibrosis, cirrhosis, and hepatocellular carcinoma, the most important clinical complication of which is periportal liver fibrosis, and there is growing evidence of significant changes in the gut microbiota profile in liver disease induced by schistosome infections [8, 18, 66]. This further illustrates the association between schistosomiasis gut microbiota and liver injury. In this section, we argue that the intestinal flora play an important role in schistosomiasis-mediated disease through the pathway of the gut–liver axis, based on the model of the gut–liver axis in other liver diseases and the interaction between the liver, intestine, and microbiota after schistosome infections. We also attempt to elaborate the process by which the intestine and liver communicate through a tight bidirectional connection of the biliary tract, portal vein, and body circulation to provide a basis for the contribution made in schistosomiasis (Fig. 2).

In recent reviews, Tripathi et al. summarize gut–liver communication in liver diseases and suggest that liver injury may be caused by excessive interactions between the gut microbiota through specific molecules (e.g., TMA, acetaldehyde, and lipopolysaccharide [LPS]) and the host immune system through Kupffer cell-mediated liver inflammation. They also explore the molecular, genetic, and microbiome relationships, and discuss the prospects for using the microbiome to determine liver disease staging and predict the effects of drugs, diet, and other interventions at the population and individual levels [67]. In 2018, Konturek et al. supplemented the understanding of the functional impairment of the intestinal mucosal barrier (“leaky gut”) and the increased bacterial translocation through the gut–liver axis, which may play an important role in the progression of liver diseases. They also highlighted the detrimental effect of gut dysbiosis in the pathogenesis and progression of chronic liver diseases such as non-alcoholic fatty liver disease (NAFLD), alcoholic liver disease (ALD), immune-mediated liver diseases, cirrhosis, and hepatocellular carcinoma. They proposed that improving the ecological imbalance of the intestinal microbiota through the use of prebiotics, probiotics, and fecal microbiota transplantation could enhance intestinal barrier function, offering a promising new approach to managing chronic liver disease [68]. In 2020, Lee et al. observed significant changes in microbiome diversity according to the severity of fibrosis in patients with NAFLD, finding not only *Ruminococcaceae* and *Veillonellaceae* as the main microbiota associated with the severity of liver fibrosis, but in addition, elevated fecal bile acids (BAs) and propionates in patients with significant liver fibrosis, adding to the picture of changes in metabolites in models of liver fibrosis and providing evidence for the role of the gut–liver axis in the pathogenesis of liver fibrosis [69]. In a 2022 study, Tilg et al. summarized the bidirectional crosstalk of liver diseases along the gut–liver axis controlling liver diseases and using environmental and host mediators. Nutrients, microbial antigens, metabolites, and BAs regulate metabolic and immune responses in the gut and liver, which mutually shape the microbial community structure and function. This perturbation of host–microbial interactions is observed in multiple experimental liver diseases and is facilitated by a compromised intestinal barrier, which fuels the intestinal–liver crosstalk in liver inflammation (NAFLD, ALD), and in cirrhosis (a common sequela of these diseases), molecular patterns associated with intestinal microbiota and microbial pathogens constitute hepatic inflammation and clinical complications such as hepatic encephalopathy [70].

The microbiota in the gut and the liver communicate through a tight bidirectional connection between the bile duct, portal vein, and body circulation (Fig. 2). The liver communicates with the intestine by releasing BAs and many bioactive mediators into the biliary tract and the body’s circulation [71]. Hepatic stellate cells (HSCs) are one of the major sources of collagen in the liver and play a crucial role in schistosomiasis-induced fibrosis [72]. The main function of resting HSCs is to store vitamin A, but in response to liver tissue injury, they are activated and transdifferentiated into myofibroblasts characterized by the production of extracellular matrix (ECM) components rich in protofibrillar collagen [73]. In mice infected with *S. japonicum*, activated HSCs were found at the margins of liver granulomas. These activated HSCs exhibited upregulation of specific markers, including junctional protein, smooth muscle actin (a-SMA), and glial fibrillary acidic protein (GFAP). Moreover, these activated HSCs may play a significant role as effector cells in the development of granuloma-associated fibrosis [74]. The same predominance of GFAP-positive cells observed in *S. mansoni*-infected mice suggests that HSCs play a major role in the deposition of connective tissue in human schistosomiasis livers [75]. Therefore, these activated HSCs in granulomas can be used as indicators of collagen deposition in hepatic schistosomiasis. In contrast, intrahepatic blastocytes (macrophages of interest) have shown that the rapid type 2 deviation in the liver after schistosome infections may be due to alterations in blastocytes that induce soluble worm antigen preparation (SWAP)-mediated type 2 development of hepatic T cells [76].

In the intestine, hosts and microorganisms metabolize BAs and amino acids, the products of which are transferred to the liver via the portal vein and affect liver function. BAs are amphiphilic molecules synthesized from cholesterol in pericentral hepatocytes. Approximately 95% of BAs are actively reabsorbed at the end of the ileum and transported back to the liver [77, 78]. The remaining 5% are uncoupled, dehydrogenated, and dehydroxylated by the colonic microbiota to form secondary BAs, which reach the liver via passive absorption into the portal circulation. The liver recovers BAs and secretes them to the biliary tract, completing the so-called enterohepatic circulation, an exchange system between the intestine and the liver. SCFAs are produced by bacteria in the colon by fermenting unabsorbed dietary fiber and provide a source of energy for metabolism in the colon. Butyrate, propionate (produced by bacterial fermentation), and acetate (produced by host and bacteria) are the main SCFAs in the large intestine. Butyrate is a source of energy for enterocytes and helps maintain the intestinal barrier [79]. Schistosomiasis-induced liver injury is thought to be marked by reduced levels of acetate, butyrate, and propionate [80]. A reduction in butyrate is associated with a weakening of intestinal tight junctions and, therefore, permeability. Choline is a macronutrient that is important for liver function, brain development, neurological function, muscle movement, and maintenance of healthy metabolism [81]. Choline is processed by the host into phosphatidylcholine (lecithin), which contributes to the excretion of very-low-density lipoprotein (VLDL) particles from the liver. This process prevents the hepatic accumulation of triglycerides (hepatic steatosis) [82]. In addition, choline can be converted to TMA by intestinal bacteria; TMA can be transferred to the liver via the portal circulation, where it is converted to trimethylamine *N*-oxide (TMAO) [83]. An increase in both TMA and TMAO can be detected in schistosomiasis [37], and the increase in TMAO is accompanied by a decrease in the level of phosphorylcholine produced by the host, an imbalance specific to patients with intestinal ecology disorders. In 2022, Lin et al. identified a series of intestinal bacterial signatures (*Mycobacterium*-like, *Braconella,* and *Enterococcus*) to distinguish schistosomiasis from liver injury and showed for the first time a significant correlation with liver granulomas, fibrosis, hydroxyproline, alanine transaminase (ALT), or aspartate transaminase (AST) levels in *S. japonicum* infection, providing further evidence for a relationship between serological detection of schistosomiasis and levels of liver injury [84]. In summary, we conclude that significant changes in the gut microbiota profile occur in *S. japonicum* infection-induced disease, and provide preliminary evidence for an association between the gut microbiota and liver injury in schistosomiasis, suggesting that the gut flora play an important role in schistosomiasis-mediated disease through the pathway provided by the gut–liver axis. However, the causal relationship between intestinal flora and schistosomiasis and the specific mechanism of intestinal flora through the gut–liver axis in schistosomiasis remain obscure and deserve further investigation.

### Probiotic treatments: an emerging therapeutic strategy in schistosomiasis?

Chemotherapy is the main method for controlling schistosomiasis, as there is no vaccine against it, and the mollusks which are intermediate hosts are not easily attacked. It is worrying to have only PZQ to treat schistosomiasis, mainly because it is not effective against schistosomula and young worms, and resistance may develop in endemic areas of high prevalence. The development of drug resistance is also a concern given the extensive repeated use of the drugs in a large number of individuals [85]. Given the limitations of PZQ, many groups have been working to evaluate new therapeutic alternatives for schistosomiasis [86–95]. Traditional treatments that target the host's gut flora include diet, antibiotics, fecal microbiota transplant (FMT), and probiotics [96]. However, diet has little effect, antibiotics are susceptible to the development of resistance, and it is critical to the efficacy and safety of FMT therapies to establish criteria for proper treatment and screening of donor feces, which are currently underdeveloped. Probiotic therapy stands out from traditional approaches [97]. Modulation of the gut microbiota through the use of probiotics is an emerging and promising treatment for gut–liver axis dysfunction and schistosomiasis infection. Probiotics are defined by the Food and Agriculture Organization of the United Nations (FAO)/WHO as live microorganisms that are beneficial to host health when administered in adequate amounts [98]. To date, various attempts have been made to study the protective and therapeutic effects of beneficial bacteria in mouse models utilized for the management of schistosomiasis, as shown in Table 3. In the case of schistosomiasis, research has been conducted on the efficacy of probiotics alone or in combination with antiparasitic drugs [88, 94].

**Table 3:** Probiotic strains used against schistosomiasis infection in mice

Pathogen	Probiotics strain	Host	Dose/route	Mechanisms	Anti-schistosomal effect	References
*S. mansoni*	*Zymomonas mobilis*	C57B1/10 male mice	Oral administration of 10^9^ CFU/ml, at a dose of 0.3 ml/day	Provoked a secondary immune response	Resulted in 61% protection	[95]
*S. mansoni*	Probiotic yogurt (containing probiotic strains of *Lactobacillus casei*, *Lactobacillus acidophilus*, *Lactobacillus plantarum *, and* Lactobacillus reutrie* strains)	Female Swiss albino mice	10^6^ CFU each mixed with feed	Significant stimulation of IgM response against SWAP before and after infection	Increased IgM; decreased activity of AST, LDH, and γGT	[91]
*S. mansoni*	Probiotic labneh containing *Streptococcus salivarius* subsp. *thermophilus, Lactobacillus delbrueckii* subsp. *bulgaricus*, and DVS-ABT2	Female Swiss albino mice	Fed probiotic labneh and garlic and onions for 21 days before and 45 days after infection	Improved intestinal balance	50–66% reduction in worm burden; 70% and 56.44% egg count reduction in liver and intestine, respectively	[89]
*S. mansoni*	*Lactobacillus sporogenes*	Male albino CD-1 mice	Oral administration of 12.5 million spores/mouse/week for 8 weeks	Decreased cytokine-induced chromosomal aberrations and DNA damage	Significant reduction in chromosomal aberrations	[94]
*S. mansoni*	*Lactobacillus sporogenes*	Male albino CD-1 mice	Oral administration of 12.5 million spores/mouse/week for 8 weeks from the first day of infection	Reduced DNA damage, ameliorated hepatic and intestinal damage	Reduced worm and egg count	[88]
*S. mansoni*	*Lactobacillus acidophilus* ATCC 4356 and *Lactobacillus delbrueckii* subsp.* bulgaricus* DSM 20080	Male CD-1 Swiss albino mice	Mixed at a ratio of 108:108 CFU/g, 100 μl/mouse through oral gavage tubes	Decreased transaminase levels in serum, reduced oxidative stress, and exhibited antioxidant properties	Revealed significant anti-apoptotic and antioxidant effects, and decreased the granuloma formation in hepatic tissue	[93]
*S. japonicum*	*Bacillus subtilis* CMCC(B) 63501	Male pathogen-free BALB/c mice	Oral administration of 0.3 ml/mouse/3 days for 6 weeks	Alleviated intestinal injury by modulating gene expression profiles	Attenuated intestinal and liver pathological injury	[87]
*S. mansoni*	*Bacillus clausii*	Swiss Webster female mice	Daily by gavage in a single dose of 2 × 109 CFU in 300 μl of sterile saline solution	Stimulated non-specific host immune resistance to pathogens	Reduced total worm load and female worms	[92]
*S. japonicum*	*Bacillus amyloliquefaciens*	Female pathogen-free BALB/c mice	Each mouse given a 0.3-ml suspension of *B. amyloliquefaciens* every 3 days	Reshaped the intestinal microenvironment of infected mice by modulating the relative abundance of potentially beneficial and harmful bacteria as well as the network of interactive relationships of the intestinal microbiome	Alleviated the pathological reaction	[86]
*S. mansoni*	*Bacillus cereus* GM	Female Swiss Webster mice	10^5^ spores in 300 μl of sterile saline solution/mouse/day	Th1 cytokines (IFN-γ, TNF, and IL-6) remained elevated, and were significantly involved in the reduction of the parasitic burden of *S. mansoni*	Reduced the number of worms and eggs in the liver tissue and the volume of granulomas, and improved the levels of some liver markers	[90]

SWAP, soluble worm antigen preparation; AST, aspartate transaminase; LDH, lactate dehydrogenase; γGT, gamma-glutamyl transferase; DVS-ABT2, containing *Streptococcus thermophilus*, *Lactobacillus acidophilus*, and *Bifidobacterium bifidum*; CFU, colony-forming units; IFN-γ, interferon gamma; TNF, tumor necrosis factor; IL-6, interleukin 6

Studies on probiotics alone have generally confirmed that probiotics are able to mitigate intestinal and liver pathological injury associated with schistosome infections. In a recent study by El-Khadragy et al., the effectiveness of a combination of *Lactobacillus acidophilus* (ATCC 4356) and *Lactobacillus delbrueckii* subsp. *bulgaricus* (DSM 20080) was assessed in a mouse model of *S. mansoni* infection. The probiotics were administered before or after parasite exposure, and the probiotic mixture was also utilized as a yogurt treatment in a saline buffer. The researchers observed a decrease in adult worm burden in mice treated with either probiotics or yogurt, both before and after infection (probiotics: 68% and 60% reduction pre- and post-infection, respectively; yogurt: 72% and 64% reduction pre- and post-infection, respectively). The probiotics demonstrated comparable efficacy to PZQ, reducing adult worm numbers by 78%. A similar trend was observed in reducing worm egg quantities in the liver. Notably, *S. mansoni* infection led to elevated levels of hepatic matrix metalloproteinase-9 (MMP-9), lipid peroxidation, and NO, while reduced glutathione levels were observed. However, these effects could be prevented or reversed by both probiotic treatment and PZQ. It is worth mentioning that PZQ failed to decrease NO levels in the liver, whereas all probiotic treatments proved successful [93]. Mice were fed probiotic yogurt (containing probiotic strains of *Lactobacillus casei*, *L. acidophilus*, *Lactobacillus plantarum*, and* Lactobacillus reutrie* strains) before and after infection with *S. mansoni*. This strategy resulted in an increase in body weight as well as a decrease in spleen and liver weight of the mice to values close to those of the controls. The probiotic yogurt was found to show immunomodulatory effects by stimulating immunoglobulin M (IgM) responses against soluble helminth antigens compared to untreated controls. Although infection increased plasma activity of AST, lactate dehydrogenase (LDH), and gamma-glutamyl transferase, the addition of the probiotic yogurt resulted in a significant reduction of these enzymes in infected animals [91]. A previous study reported that *Bacillus subtilis* could improve digestive health by strengthening the intestinal barrier and limiting the inflammatory response, helping to limit the intestinal inflammatory response and homeostasis in mice [99]. And in a recent study, it was revealed for the first time that *B. subtilis* attenuated liver and intestinal damage in *S. japonicum*-infected mice, implying that probiotic supplementation may contribute to host biology during schistosomiasis [87]. Furthermore, a recent study showed that the use of *Bacillus amyloliquefaciens* was effective in treating and alleviating the symptoms of *S. japonicum*, where the early establishment of probiotic colonization in mice infected with *S. japonicum* reduced liver and intestinal fibrosis and granulomas in the acute phase. At the same time, it was able to reshape the intestinal microenvironment of infected mice by modulating the relative abundance of potentially beneficial and harmful bacteria and the network of interactions between the intestinal microbiota [86, 100]. In addition, a Brazilian study conducted by dos Santos et al. found that the burden of *S. mansoni*-infected mice was reduced with *Bacillus cereus* GM by modulating the immune response. For example, it reduced the number of worms, the number of eggs in the liver tissue, and the volume of granulomas, and increased the levels of some liver markers. These results indicate the possibility of using the probiotic *B. cereus* GM in the treatment of schistosomiasis, mainly in endemic areas where the population is at risk of persistent infection [90].

The combination of probiotics with antischistosomal drugs has also shown significant effects. In 2012, Zowail et al. investigated the treatment of mice previously infected with *S. mansoni* using *Bacillus coagulans*, PZQ, or a combination of the two. The results showed a 53% reduction in the number of adult worms with probiotics alone, an 89% reduction with standard PZQ treatment, and 100% reduction with the combination treatment [94]. As demonstrated by EI-Esawy et al., *S. mansoni* infection and PZQ treatment can lead to chromosomal aberrations and DNA damage. To mitigate this effect, *B. coagulans* was used as a supplement to antiparasitic treatment. When PZQ treatment was combined with *B. coagulans*, a significant reduction in chromosomal aberrations induced by infection or PZQ treatment was observed [88]. These findings highlight the importance of evaluating probiotics as adjuncts to conventional antischistosomal therapy.

The findings from these experiments suggest that certain strains of *Lactobacillus* and *Bacillus* genera have the potential to serve as probiotics for the treatment of schistosomiasis. The mode of action of these probiotics can be strain-specific or a combination of different mechanisms. They may involve one or more modes of action, including production of antimicrobial substances, modulation of the mucosal immune system, modification of the gut microbial community, and enhancement of enzymatic activity [101]. Specifically, we propose various mechanisms to explain the main modes of action of probiotics against schistosomiasis, as follows: (1) Immunostimulation and immunomodulation by components of the innate or adaptive immune system are the main mechanisms through which probiotics act against schistosomiasis. This includes (i) modulation of cytokine expression to reduce the burden of schistosomiasis, (ii) counteracting cytokine-induced apoptosis by decreasing chromosomal aberrations and DNA damage, and (iii) enhancing the humoral immune responses, thereby promoting an immune barrier in the gut. (2) Probiotics increase the population of beneficial microorganisms by enhancing the intestinal barrier and regulating the balance of the intestinal microbial community, such as *Lactobacillus* and *Bifidobacterium*, which then inhibit the growth of harmful pathogens by competing for attachment sites in the intestinal mucosa. (3) The secretion of antimicrobial substances (e.g., bacteriocins) and organic acids (e.g., lactic, acetic, and butyric acids), which are mainly secreted by species of *Lactobacillus*, may have a larvicidal effect on schistosomiasis. (4) Probiotics exhibit antioxidant properties through scavenging of reactive oxygen species (ROS) and activation of superoxide dismutase (SOD) and catalase (CAT) to reduce oxidative stress. It is worth noting that most of the effects observed in relation to probiotics and schistosomes have been demonstrated in animal experiments and in vitro cultures, with limited reports on human trials. Additionally, the molecular mechanisms underlying these beneficial microbial actions remain poorly understood. It seems unreasonable to propose probiotics as an alternative to classical therapies such as drugs or vaccines against schistosomes, while their use in reducing the risk of infection or as complementary therapies to classical treatments seems more realistic and relevant. Research on the role of probiotics against schistosomes is still in its infancy, and further studies on host–microbe or schistosome–microbe interactions using state-of-the-art immunogenetic techniques, genetic engineering, and synthetic biology may elucidate the mechanisms of probiotic action against schistosomiasis.

## Conclusions 

Because of the complicated transmission routes and many influencing factors of schistosomiasis, the disease is difficult to prevent and control [102]. WHO estimates show that at least 251.4 million people need preventive treatment, which should be repeated over several years, and 78 countries have reported schistosomiasis transmission [4]. In the last decade, studies of schistosome–microbiome interactions have increasingly included models of schistosomiasis (Table 1). In particular, high-throughput amplicon sequencing techniques for large-scale rapid characterization of complex bacterial communities have been utilized to uncover the effects of schistosome infections on the host. Based on the altered microbial profiles, it is reasonable to speculate that the host damage from schistosome infections may be transmitted by intestinal microorganisms through the gut–liver axis. Schistosome infections lead to intestinal granulomatous and fibrotic lesions, and the dysbiosis of the intestinal flora increases toxic metabolites, leading to inflammatory damage in the liver, exacerbating hepatic lesions, and facilitating the development of hepatic fibrosis, which can have a serious impact on the patients' prognosis.

Following schistosome infections, Firmicutes and Proteobacteria were the most responsive phyla in schistosomiasis [18, 20–22]. A significant decrease in the relative abundance of *Comamonas* and *Psychrobacter* in Firmicutes [18] suggests that schistosome infections may induce a systemic inflammatory response through potentially efficient xenobiotic-metabolizing species. An increase in the relative abundance of *Turicibacter* in Proteobacteria [18] suggests that it may be involved in the development of systemic inflammation through alterations in immune cell activation. However, the relative abundance of *Clostridium* correlated with total cholesterol levels was decreased in infected hosts [28], suggesting that these taxa may play a role in infection by influencing lipid metabolism. *Butyricimonas* produces butyrate, which is a source of energy for intestinal cells and contributes to the maintenance of the intestinal barrier [29]; the decreased abundance of *Butyricimonas* suggests that the host's intestinal barrier is disrupted. Thus, changes in the relative abundance of these bacteria suggest that the microbiota is associated with changes in metabolism and immune responses during schistosome infections.

It has been shown that microbiota-based probiotic modulation targeting the gut–liver axis is a promising and beneficial therapeutic approach. Probiotic supplementation of schistosome-infected hosts showed significant anti-apoptotic and antioxidant effects, reducing the number of eggs and the volume of granulomas in liver tissues of schistosome-infected mice and increasing the levels of some liver markers. The results of these experiments suggest that some bacterial strains in *Lactobacillus* and *Enterococcus* could be used as probiotics for the prevention or treatment of schistosomiasis after validation in replicated animal clinical trials. Their mode of action can be strain-specific or a combination of different mechanisms. In addition, the mechanisms of probiotics when used as adjunctive therapies in combination with conventional antischistosomal drugs to alleviate disease symptoms and/or mitigate the side effects of conventional antischistosomal drugs should be further elucidated, in particular their response to interactions with the intestinal microbiota and their effect on the bioavailability, bioactivity, and toxicity of the medication. A better understanding of the molecular mechanisms underlying the beneficial effects of probiotics on schistosome infections is essential to validating the approach. Therefore, more in-depth studies are needed, including extended clinical studies, and using more defined protocols with specific probiotics and experimental models. For example, is the optimal time to start treatment before or after the onset of infection? What is the optimal duration of treatment? How can probiotic strains and antischistosomal drugs be combined to maximize treatment efficiency? Can probiotic strains be used as an effective strategy to prevent schistosomiasis? Moreover, the design of clinical trials with placebo controls is critical, as these treatments are expected to be applied to human or animals.

Given the complexity of the relationship and mechanisms of the interactions between schistosome infections and the intestinal flora, comprehensive and in-depth studies combining macrogenomics, macrotranscriptomics, macrometabolomics, proteomics, and immunomics are also needed to unearth more information about the key small molecules that undergo changes during schistosome infections, in order to elucidate the mechanisms and applications of microorganisms in schistosomiasis. As the role of the microbiota in the development, prognosis, and treatment of schistosomiasis is increasingly recognized, further development of synthetic biology and optimization of existing molecular tools will also be key for targeted, stable, and safe modifications of the composition and function of the gut microbiota. Integrating the knowledge gained in these two research areas will make it possible to fully utilize the potential of microbial-based interventions for schistosomiasis and translate them into new and reliable strategies for the management and control of schistosomiasis.

## Data Availability

All data regarding this study are included in the manuscript.

## References

[1] McManus DP, Dunne DW, Sacko M, Utzinger J, Vennervald BJ, Zhou X-N. Schistosomiasis. Nat Rev Dis Primer. 2018;4:13. 10.1038/s41572-018-0013-8 30093684

[2] Barnett R. Schistosomiasis. Lancet Lond Engl. 2018;392:2431. 10.1016/S0140-6736(18)33008-3 30527408

[3] Colley DG, Bustinduy AL, Secor WE, King CH. Human schistosomiasis. Lancet Lond Engl. 2014;383:2253–64. 10.1016/S0140-6736(13)61949-2 PMC4672382 24698483

[4] Schistosomiasis. https://www.who.int/news-room/fact-sheets/detail/schistosomiasis. Accessed 12 Dec 2023.

[5] Comin F, Speziali E, Correa-Oliveira R, Faria AMC. Aging and immune response in chronic human schistosomiasis. Acta Trop. 2008;108:124–30. 18582841 10.1016/j.actatropica.2008.05.004

[6] Carson JP, Ramm GA, Robinson MW, McManus DP, Gobert GN. Schistosome-induced fibrotic disease: the role of hepatic stellate cells. Trends Parasitol. 2018;34:524–40. 29526403 10.1016/j.pt.2018.02.005

[7] Sender R, Fuchs S, Milo R. Revised estimates for the number of human and bacteria cells in the body. PLoS Biol. 2016;14:e1002533. 27541692 10.1371/journal.pbio.1002533 PMC4991899

[8] Zhao Y, Yang S, Li B, Li W, Wang J, Chen Z, et al. Alterations of the mice gut microbiome via *Schistosoma japonicum* ova-induced granuloma. Front Microbiol. 2019;10:352. 30891012 10.3389/fmicb.2019.00352 PMC6411663

[9] Zhang D, Hu Q, Liu X, Liu X, Gao F, Liang Y, et al. A longitudinal study reveals the alterations of the *Microtus fortis *colonic microbiota during the natural resistance to *Schistosoma japonicum* infection. Exp Parasitol. 2020;219:108030. 33080305 10.1016/j.exppara.2020.108030

[10] Holzscheiter M, Layland LE, Loffredo-Verde E, Mair K, Vogelmann R, Langer R, et al. Lack of host gut microbiota alters immune responses and intestinal granuloma formation during schistosomiasis. Clin Exp Immunol. 2014;175:246–57. 24168057 10.1111/cei.12230 PMC3892416

[11] Nogueira RA, Lira MGS, Licá ICL, Frazão GCCG, Dos Santos VAF, Filho ACCM, et al. Praziquantel: An update on the mechanism of its action against schistosomiasis and new therapeutic perspectives. Mol Biochem Parasitol. 2022;252:111531. 36375598 10.1016/j.molbiopara.2022.111531

[12] Zwang J, Olliaro P. Efficacy and safety of praziquantel 40 mg/kg in preschool-aged and school-aged children: a meta-analysis. Parasit Vectors. 2017;10:47. 28126024 10.1186/s13071-016-1958-7 PMC5270314

[13] Wu K, Zhai X, Huang S, Jiang L, Yu Z, Huang J. Protein kinases: potential drug targets against *Schistosoma japonicum*. Front Cell Infect Microbiol. 2021;11:691757. 34277472 10.3389/fcimb.2021.691757 PMC8282181

[14] Maurice CF, Knowles SCL, Ladau J, Pollard KS, Fenton A, Pedersen AB, et al. Marked seasonal variation in the wild mouse gut microbiota. ISME J. 2015;9:2423–34. 26023870 10.1038/ismej.2015.53 PMC4611506

[15] Pickard JM, Zeng MY, Caruso R, Núñez G. Gut microbiota: role in pathogen colonization, immune responses, and inflammatory disease. Immunol Rev. 2017;279:70–89. 28856738 10.1111/imr.12567 PMC5657496

[16] Carding S, Verbeke K, Vipond DT, Corfe BM, Owen LJ. Dysbiosis of the gut microbiota in disease. Microb Ecol Health Dis. 2015;26:26191. 25651997 10.3402/mehd.v26.26191 PMC4315779

[17] Zhang B, Wu X, Song Q, Ning A, Liang J, Song L, et al. Gut microbiota modulates intestinal pathological injury in *Schistosoma japonicum*-infected mice. Front Med. 2020;7:588928. 10.3389/fmed.2020.588928 PMC7703745 33313045

[18] Jiang Y, Yuan Z, Shen Y, Rosa BA, Martin J, Cao S, et al. Alteration of the fecal microbiota in Chinese patients with *Schistosoma japonicum* infection. Parasite Paris Fr. 2021;28:1. 10.1051/parasite/2020074 PMC7792497 33416489

[19] Fricke WF, Song Y, Wang A-J, Smith A, Grinchuk V, Mongodin E, et al. Type 2 immunity-dependent reduction of segmented filamentous bacteria in mice infected with the helminthic parasite *Nippostrongylus brasiliensis*. Microbiome. 2015;3:40. 26377648 10.1186/s40168-015-0103-8 PMC4574229

[20] Jenkins TP, Peachey LE, Ajami NJ, MacDonald AS, Hsieh MH, Brindley PJ, et al. *Schistosoma mansoni* infection is associated with quantitative and qualitative modifications of the mammalian intestinal microbiota. Sci Rep. 2018;8:12072. 30104612 10.1038/s41598-018-30412-x PMC6089957

[21] Floudas A, Aviello G, Schwartz C, Jeffery IB, O’Toole PW, Fallon PG. *Schistosoma mansoni* worm infection regulates the intestinal microbiota and susceptibility to colitis. Infect Immun. 2019;87:e00275-e319. 31138616 10.1128/IAI.00275-19 PMC6652750

[22] Ajibola O, Rowan AD, Ogedengbe CO, Mshelia MB, Cabral DJ, Eze AA, et al. Urogenital schistosomiasis is associated with signatures of microbiome dysbiosis in Nigerian adolescents. Sci Rep. 2019;9:829. 30696838 10.1038/s41598-018-36709-1 PMC6351658

[23] Osakunor DNM, Munk P, Mduluza T, Petersen TN, Brinch C, Ivens A, et al. The gut microbiome but not the resistome is associated with urogenital schistosomiasis in preschool-aged children. Commun Biol. 2020;3:155. 32242065 10.1038/s42003-020-0859-7 PMC7118151

[24] Shin N-R, Whon TW, Bae J-W. Proteobacteria: microbial signature of dysbiosis in gut microbiota. Trends Biotechnol. 2015;33:496–503. 26210164 10.1016/j.tibtech.2015.06.011

[25] Litvak Y, Byndloss MX, Tsolis RM, Bäumler AJ. Dysbiotic Proteobacteria expansion: a microbial signature of epithelial dysfunction. Curr Opin Microbiol. 2017;39:1–6. 28783509 10.1016/j.mib.2017.07.003

[26] Gogleva AA, Kaparullina EN, Doronina NV, Trotsenko YA. *Methylophilus flavus* sp. nov. and *Methylophilus luteus* sp. nov., aerobic, methylotrophic bacteria associated with plants. Int J Syst Evol Microbiol. 2010;60:2623–8. 20023062 10.1099/ijs.0.019455-0

[27] Gülden E, Chao C, Tai N, Pearson JA, Peng J, Majewska-Szczepanik M, et al. TRIF deficiency protects non-obese diabetic mice from type 1 diabetes by modulating the gut microbiota and dendritic cells. J Autoimmun. 2018;93:57–65. 29960834 10.1016/j.jaut.2018.06.003 PMC6108920

[28] Gózd-Barszczewska A, Kozioł-Montewka M, Barszczewski P, Młodzińska A, Humińska K. Gut microbiome as a biomarker of cardiometabolic disorders. Ann Agric Environ Med. 2017;24:416–22. 28954482 10.26444/aaem/75456

[29] Gong J, Qiu W, Zeng Q, Liu X, Sun X, Li H, et al. Lack of short-chain fatty acids and overgrowth of opportunistic pathogens define dysbiosis of neuromyelitis optica spectrum disorders: A Chinese pilot study. Mult Scler J. 2019;25:1316–25. 10.1177/1352458518790396 30113252

[30] He X, McLean JS, Edlund A, Yooseph S, Hall AP, Liu S-Y, et al. Cultivation of a human-associated TM7 phylotype reveals a reduced genome and epibiotic parasitic lifestyle. Proc Natl Acad Sci. 2015;112:244–9. 25535390 10.1073/pnas.1419038112 PMC4291631

[31] Hu Y, Chen J, Xu Y, Zhou H, Huang P, Ma Y, et al. Alterations of gut microbiome and metabolite profiling in mice infected by *Schistosoma japonicum*. Front Immunol. 2020;11:569727. 33162984 10.3389/fimmu.2020.569727 PMC7580221

[32] Jandhyala SM. Role of the normal gut microbiota. World J Gastroenterol. 2015;21:8787. 26269668 10.3748/wjg.v21.i29.8787 PMC4528021

[33] Bloom SM, Bijanki VN, Nava GM, Sun L, Malvin NP, Donermeyer DL, et al. Commensal Bacteroides species induce colitis in host-genotype-specific fashion in a mouse model of inflammatory bowel disease. Cell Host Microbe. 2011;9:390–403. 21575910 10.1016/j.chom.2011.04.009 PMC3241010

[34] Zhang Y, Wang Y, Jiang Y, Pan W, Liu H, Yin J, et al. T follicular helper cells in patients with acute schistosomiasis. Parasit Vectors. 2016;9:321. 27266984 10.1186/s13071-016-1602-6 PMC4895967

[35] Dubourg G, Lagier J-C, Armougom F, Robert C, Audoly G, Papazian L, et al. High-level colonisation of the human gut by *Veillonellaceae* following broad-spectrum antibiotic treatment. Int J Antimicrob Agents. 2013;41:149–55. 23294932 10.1016/j.ijantimicag.2012.10.012

[36] Morrison DJ, Preston T. Formation of short chain fatty acids by the gut microbiota and their impact on human metabolism. Gut Microbes. 2016;7:189–200. 26963409 10.1080/19490976.2015.1134082 PMC4939913

[37] Wang Y, Holmes E, Nicholson JK, Cloarec O, Chollet J, Tanner M, et al. Metabonomic investigations in mice infected with *Schistosoma mansoni*: an approach for biomarker identification. Proc Natl Acad Sci. 2004;101:12676–81. 15314235 10.1073/pnas.0404878101 PMC515115

[38] Li JV, Holmes E, Saric J, Keiser J, Dirnhofer S, Utzinger J, et al. Metabolic profiling of a *Schistosoma mansoni* infection in mouse tissues using magic angle spinning-nuclear magnetic resonance spectroscopy. Int J Parasitol. 2009;2009:1. 10.1016/j.ijpara.2008.10.010 19068218

[39] Garcia-Perez I, Couto Alves A, Angulo S, Li JV, Utzinger J, Ebbels TMD, et al. Bidirectional correlation of NMR and capillary electrophoresis fingerprints: a new approach to investigating *Schistosoma mansoni* infection in a mouse model. Anal Chem. 2010;82:203–10. 19961175 10.1021/ac901728w

[40] Garcia-Perez I, Angulo S, Utzinger J, Holmes E, Legido-Quigley C, Barbas C. Chemometric and biological validation of a capillary electrophoresis metabolomic experiment of *Schistosoma mansoni* infection in mice. Electrophoresis. 2010;31:2338–48. 20583011 10.1002/elps.200900523

[41] Balog CIA, Vennervald BJ, Mayboroda OA, Deelder AM. Metabonomic investigation of human *Schistosoma mansoni* infection. Mol Biosyst. 2011;7:1473–80. 21336380 10.1039/c0mb00262c

[42] Wang Y, Utzinger J, Xiao S-H, Xue J, Nicholson JK, Tanner M, et al. System level metabolic effects of a *Schistosoma japonicum* infection in the Syrian hamster. Mol Biochem Parasitol. 2006;146:1–9. 16337285 10.1016/j.molbiopara.2005.10.010

[43] Wu J, Xu W, Ming Z, Dong H, Tang H, Wang Y. Metabolic changes reveal the development of schistosomiasis in mice. PLoS Negl Trop Dis. 2010;4:e807. 20824219 10.1371/journal.pntd.0000807 PMC2930859

[44] Huang Y, Wu Q, Zhao L, Xiong C, Xu Y, Dong X, et al. UHPLC-MS-based metabolomics analysis reveals the process of schistosomiasis in mice. Front Microbiol. 2020;11:1517. 32760365 10.3389/fmicb.2020.01517 PMC7371968

[45] Zhu X, Chen L, Wu J, Tang H, Wang Y. *Salmonella typhimurium* infection reduces Schistosoma japonicum worm burden in mice. Sci Rep. 2017;7:1349. 28465515 10.1038/s41598-017-00992-1 PMC5430953

[46] Wang Y, Holmes E, Nicholson JK, Cloarec O, Chollet J, Tanner M, et al. Metabonomic investigations in mice infected with *Schistosoma mansoni*: an approach for biomarker identification. Proc Natl Acad Sci USA. 2004;101:12676–81. 15314235 10.1073/pnas.0404878101 PMC515115

[47] Lambertucci JR, Serufo JC, Gerspacher-Lara R, Rayes AAM, Teixeira R, Nobre V, et al. *Schistosoma mansoni*: assessment of morbidity before and after control. Acta Trop. 2000;77:101–9. 10996126 10.1016/s0001-706x(00)00124-8

[48] Hambrook JR, Hanington PC. Immune evasion strategies of schistosomes. Front Immunol. 2020;11:624178. 33613562 10.3389/fimmu.2020.624178 PMC7889519

[49] Reddick LE, Alto NM. Bacteria fighting back: how pathogens target and subvert the host innate immune system. Mol Cell. 2014;54:321–8. 24766896 10.1016/j.molcel.2014.03.010 PMC4023866

[50] Muniz-Junqueira MI, Tosta CE, Prata A. Salmonelose septicêmica prolongada associada à esquistossomose: evolução do conhecimento e mecanismos imunopatogênicos. Rev Soc Bras Med Trop. 2009;42:436–45. 19802482 10.1590/s0037-86822009000400015

[51] Abruzzi A, Fried B. Coinfection of *Schistosoma* (Trematoda) with Bacteria, Protozoa and Helminths. In: Advances in parasitology. London: Elsevier; 2011. p. 1–85. 10.1016/B978-0-12-391429-3.00005-8 22137582

[52] Barnhill AE, Novozhilova E, Day TA, Carlson SA. *Schistosoma*-associated *Salmonella *resist antibiotics via specific fimbrial attachments to the flatworm. Parasit Vectors. 2011;4:1–8. 21711539 10.1186/1756-3305-4-123 PMC3143092

[53] Tallima H, El Ridi R. Increased hepatic interleukin-1, arachidonic acid, and reactive oxygen species mediate the protective potential of peptides shared by gut cysteine peptidases against *Schistosoma mansoni* infection in mice. PLoS Negl Trop Dis. 2023;17:e0011164. 36920999 10.1371/journal.pntd.0011164 PMC10042345

[54] Cherian S, Burgner DP, Cook AG, Sanfilippo FM, Forbes DA. Associations between *Helicobacter pylori* infection, co-morbid infections, gastrointestinal symptoms, and circulating cytokines in African children. Helicobacter. 2010;15:88–97. 20402811 10.1111/j.1523-5378.2009.00740.x

[55] Du Y, Agnew A, Ye X, Robinson PA, Forman D, Crabtree JE. *Helicobacter pylori* and *Schistosoma japonicum* co-infection in a Chinese population: helminth infection alters humoral responses to *H. pylori* and serum pepsinogen I/II ratio. Microbes Infect. 2006;8:52–60. 16260169 10.1016/j.micinf.2005.05.017

[56] Bhattacharjee S, Mejías-Luque R, Loffredo-Verde E, Toska A, Flossdorf M, Gerhard M, et al. Concomitant infection of *S. mansoni* and *H. pylori* promotes promiscuity of antigen-experienced cells and primes the liver for a lower fibrotic response. Cell Rep. 2019;28:231-244.e5. 31269443 10.1016/j.celrep.2019.05.108

[57] Mechanism of interaction of *Salmonella* and *Schistosoma* species. 10.1128/iai.44.2.274-281.1984. Accessed 28 Nov 2023. 10.1128/iai.44.2.274-281.1984 PMC263513 6143728

[58] *Salmonella typhimurium* infection reduces *Schistosoma japonicum* worm burden in mice. Scientific Reports. https://www.nature.com/articles/s41598-017-00992-1. Accessed 28 Nov 2023. 10.1038/s41598-017-00992-1 PMC5430953 28465515

[59] Jenkins TP, Brindley PJ, Gasser RB, Cantacessi C. Helminth microbiomes—A hidden treasure trove? Trends Parasitol. 2019;35:13–22. 30503365 10.1016/j.pt.2018.10.007

[60] Gobert GN, McManus DP, McMullan G, Creevey CJ, Carson J, Jones MK, et al. Adult schistosomes have an epithelial bacterial population distinct from the surrounding mammalian host blood. PLoS ONE. 2022;17:e0263188. 35085360 10.1371/journal.pone.0263188 PMC8794206

[61] Finlay BB, McFadden G. Anti-immunology: evasion of the host immune system by bacterial and viral pathogens. Cell. 2006;124:767–82. 16497587 10.1016/j.cell.2006.01.034

[62] The ultrastructural architecture of the adult *Schistosoma japonicum* tegument. ScienceDirect. https://www.sciencedirect.com/science/article/pii/S0020751903002558?via%3Dihub. Accessed 28 Nov 2023. 10.1016/s0020-7519(03)00255-8 14636672

[63] Formenti F, Cortés A, Deiana M, Salter S, Parkhill J, Berriman M, et al. The human blood fluke, *Schistosoma mansoni*, harbors bacteria throughout the parasite’s life cycle. J Infect Dis. 2023;228:1299–303. 37487539 10.1093/infdis/jiad288 PMC10629713

[64] Sotillo J, Pearson M, Becker L, Mulvenna J, Loukas A. A quantitative proteomic analysis of the tegumental proteins from *Schistosoma mansoni* schistosomula reveals novel potential therapeutic targets. Int J Parasitol. 2015;45:505–16. 25910674 10.1016/j.ijpara.2015.03.004

[65] Manipulation of host and parasite microbiotas: Survival strategies during chronic nematode infection. Science Advances. 10.1126/sciadv.aap7399. Accessed 28 Nov 2023. 10.1126/sciadv.aap7399 PMC5851687 29546242

[66] Cortés A, Clare S, Costain A, Almeida A, McCarthy C, Harcourt K, et al. Baseline gut microbiota composition is associated with *Schistosoma mansoni* infection burden in rodent models. Front Immunol. 2020;11:593838. 33329584 10.3389/fimmu.2020.593838 PMC7718013

[67] Tripathi A, Debelius J, Brenner DA, Karin M, Loomba R, Schnabl B, et al. The gut–liver axis and the intersection with the microbiome. Nat Rev Gastroenterol Hepatol. 2018;15:397–411. 29748586 10.1038/s41575-018-0011-z PMC6319369

[68] Konturek PC, Harsch IA, Konturek K, Schink M, Konturek T, Neurath MF, et al. Gut-liver axis: How do gut bacteria influence the liver? Med Sci. 2018;6:79. 10.3390/medsci6030079 PMC6165386 30227645

[69] Lee G, You HJ, Bajaj JS, Joo SK, Yu J, Park S, et al. Distinct signatures of gut microbiome and metabolites associated with significant fibrosis in non-obese NAFLD. Nat Commun. 2020;11:4982. 33020474 10.1038/s41467-020-18754-5 PMC7536225

[70] Tilg H, Adolph TE, Trauner M. Gut-liver axis: pathophysiological concepts and clinical implications. Cell Metab. 2022;34:1700–18. 36208625 10.1016/j.cmet.2022.09.017

[71] Stärkel P, Schnabl B. Bidirectional communication between liver and gut during alcoholic liver disease. Semin Liver Dis. 2016;36:331–9. 27997973 10.1055/s-0036-1593882

[72] Booth M, Mwatha JK, Joseph S, Jones FM, Kadzo H, Ireri E, et al. Periportal fibrosis in human *Schistosoma mansoni* infection is associated with low IL-10, Low IFN-γ, High TNF-α, or Low RANTES, depending on age and gender. J Immunol. 2004;172:1295–303. 14707108 10.4049/jimmunol.172.2.1295

[73] Friedman SL. Mechanisms of hepatic fibrogenesis. Gastroenterology. 2008;134:1655–69. 18471545 10.1053/j.gastro.2008.03.003 PMC2888539

[74] Bartley P, Ramm G, Jones M, Ruddell R, Li Y, Mcmanus D. A contributory role for activated hepatic stellate cells in the dynamics of *Schistosoma japonicum* egg-induced fibrosis. Int J Parasitol. 2006;36:993–1001. 16806222 10.1016/j.ijpara.2006.04.015

[75] Chang D, Ramalho LNZ, Ramalho FS, Martinelli ALC, Zucoloto S. Hepatic stellate cells in human schistosomiasis mansoni: a comparative immunohistochemical study with liver cirrhosis. Acta Trop. 2006;97:318–23. 16473318 10.1016/j.actatropica.2005.12.006

[76] Hayashi N, Matsui K, Tsutsui H, Osada Y, Mohamed RT, Nakano H, et al. Kupffer cells from *Schistosoma mansoni*-infected mice participate in the prompt type 2 differentiation of hepatic T cells in response to worm antigens. J Immunol. 1999;163:6702–11. 10586067

[77] Arab JP, Karpen SJ, Dawson PA, Arrese M, Trauner M. Bile acids and nonalcoholic fatty liver disease: Molecular insights and therapeutic perspectives. Hepatology. 2017;65:350–62. 27358174 10.1002/hep.28709 PMC5191969

[78] Wahlström A, Sayin SI, Marschall H-U, Bäckhed F. Intestinal crosstalk between bile acids and microbiota and its impact on host metabolism. Cell Metab. 2016;24:41–50. 27320064 10.1016/j.cmet.2016.05.005

[79] Wächtershäuser A, Stein J. Rationale for the luminal provision of butyrate in intestinal diseases. Eur J Nutr. 2000;39:164–71. 11079736 10.1007/s003940070020

[80] Wu J-F, Holmes E, Xue J, Xiao S-H, Singer BH, Tang H-R, et al. Metabolic alterations in the hamster co-infected with *Schistosoma japonicum* and *Necator americanus*. Int J Parasitol. 2010;40:695–703. 19951707 10.1016/j.ijpara.2009.11.003

[81] Zeisel SH, da Costa K-A. Choline: an essential nutrient for public health. Nutr Rev. 2009;67:615–23. 19906248 10.1111/j.1753-4887.2009.00246.x PMC2782876

[82] Mehedint MG, Zeisel SH. Choline’s role in maintaining liver function: new evidence for epigenetic mechanisms. Curr Opin Clin Nutr Metab Care. 2013;16:339–45. 23493015 10.1097/MCO.0b013e3283600d46 PMC3729018

[83] Velasquez MT, Ramezani A, Manal A, Raj DS. Trimethylamine N-oxide: the good, the bad and the unknown. Toxins. 2016;8:326. 27834801 10.3390/toxins8110326 PMC5127123

[84] Lin D, Song Q, Liu J, Chen F, Zhang Y, Wu Z, et al. Potential gut microbiota features for non-invasive detection of schistosomiasis. Front Immunol. 2022;13:941530. 35911697 10.3389/fimmu.2022.941530 PMC9330540

[85] Cioli D, Pica-Mattoccia L, Basso A, Guidi A. Schistosomiasis control: Praziquantel forever? Mol Biochem Parasitol. 2014;195:23–9. 24955523 10.1016/j.molbiopara.2014.06.002

[86] Chen H, Sun R, Wang J, Yao S, Batool SS, Yu Z, et al. *Bacillus amyloliquefaciens* alleviates the pathological injuries in mice infected with *Schistosoma japonicum* by modulating intestinal microbiome. Front Cell Infect Microbiol. 2023;13:1172298. 37265494 10.3389/fcimb.2023.1172298 PMC10230073

[87] Lin D, Song Q, Zhang Y, Liu J, Chen F, Du S, et al. *Bacillus subtilis* attenuates hepatic and intestinal injuries and modulates gut microbiota and gene expression profiles in mice infected with *Schistosoma japonicum*. Front Cell Dev Biol. 2021;9:766205. 34869360 10.3389/fcell.2021.766205 PMC8635066

[88] Mohamed AH, Osman GY, Zowail MEM, El-Esawy HMI. Effect of *Lactobacillus sporogenes* (probiotic) on certain parasitological and molecular aspects in *Schistosoma mansoni* infected mice. J Parasit Dis Off Organ Indian Soc Parasitol. 2016;40:823–32. 10.1007/s12639-014-0586-4 PMC4996199 27605791

[89] Abdel-Sala AM, Ammar N, Abdel-Hami AZ. Effectiveness of probiotic Labneh supplemented with garlic or onion oil against *Schistosoma mansoni* in infected mice. Int J Dairy Sci. 2008;3:97–104.

[90] dos Santos VHB, de Azevedo Ximenes ECP, de Souza RAF, da Silva RPC, da Conceição SM, de Andrade LVM, et al. Effects of the probiotic *Bacillus cereus* GM on experimental schistosomiasis mansoni. Parasitol Res. 2023;123:72. 38148420 10.1007/s00436-023-08090-0

[91] Ghanem K, Abdel-Salam A, Magharby AS. Immunoprophylactic effect of probiotic yoghurt feeding on *Schistosoma mansoni*-infected mice. Pol J Food Nutr Sci. 2005;2005:1.

[92] Cruz CS, França WWM, de Arújo HDA, Ximenes ECPA, de Souza VM, Albuquerque MCPA, et al. In vitro and in vivo evaluation of *Bacillus clausii* against *Schistosoma mansoni*. Acta Trop. 2022;235:106669. 36037981 10.1016/j.actatropica.2022.106669

[93] El-Khadragy MF, Al-Olayan EM, Elmallah MIY, Alharbi AM, Yehia HM, Abdel Moneim AE. Probiotics and yogurt modulate oxidative stress and fibrosis in livers of *Schistosoma mansoni*-infected mice. BMC Complement Altern Med. 2019;19:3. 30606163 10.1186/s12906-018-2406-3 PMC6318950

[94] Zowail MEM, Osman GY, Mohamed AH, El-Esawy HMI. Protective role of *Lactobacillus sporogenes* (probiotic) on chromosomal aberrations and DNA fragmentation in *Schistosoma mansoni* infected mice. Egypt Soc Exp Biol. 2012;2012:1.

[95] de Santos JFM, Vasconcelos J, de Souza JR, de Coutinho EM, Montenegro SML, Azevedo-Ximenes E. The effect of *Zymomonas mobilis* culture on experimental *Schistosoma mansoni* infection. Rev Soc Bras Med Trop. 2004;37:502–4. 15765603 10.1590/s0037-86822004000600015

[96] Zuo Z, Zhao F. Gut microbiota-targeted interventions: from conventional approaches to genetic engineering. Sci Bull. 2023;68:1231–4. 10.1016/j.scib.2023.05.018 37258375

[97] Wargo JA. Modulating gut microbes. Science. 2020;369:1302–3. 32913089 10.1126/science.abc3965

[98] Binda S, Hill C, Johansen E, Obis D, Pot B, Sanders ME, et al. Criteria to qualify microorganisms as “Probiotic” in foods and dietary supplements. Front Microbiol. 2020;11:1662. 32793153 10.3389/fmicb.2020.01662 PMC7394020

[99] Rhayat L, Maresca M, Nicoletti C, Perrier J, Brinch KS, Christian S, et al. Effect of *Bacillus subtilis* strains on intestinal barrier function and inflammatory response. Front Immunol. 2019;10:564. 30984172 10.3389/fimmu.2019.00564 PMC6449611

[100] Chen H, Huang S, Zhao Y, Sun R, Wang J, Yao S, et al. Metagenomic analysis of the intestinal microbiome reveals the potential mechanism involved in *Bacillus amyloliquefaciens* in treating schistosomiasis japonica in mice. Microbiol Spectr. 2024;5:e0373523. 10.1128/spectrum.03735-23. 38441977 10.1128/spectrum.03735-23 PMC10986500

[101] Bajagai YS, Klieve A, Dart P, Bryden W. Probiotics in animal nutrition: production, impact and regulation. 2016.

[102] Lü S, Lü C, Li YL, Xu J, Hong QB, Zhou J, et al. Expert consensus on the strategy and measures to interrupt the transmission of schistosomiasis in China. Zhongguo Xue Xi Chong Bing Fang Zhi Za Zhi Chin J Schistosomiasis Control. 2021;33:10–4. 10.16250/j.32.1374.2021007 33660468

[103] Kay GL, Millard A, Sergeant MJ, Midzi N, Gwisai R, Mduluza T, et al. Differences in the faecal microbiome in *Schistosoma haematobium* infected children vs. uninfected children. PLoS Negl Trop Dis. 2015;9:e0003861. 26114287 10.1371/journal.pntd.0003861 PMC4482744

[104] Zhou C, Li J, Guo C, Zhou Z, Yang Z, Zhang Y, et al. Alterations in gut microbiome and metabolite profile of patients with *Schistosoma japonicum* infection. Parasit Vectors. 2023;16:346. 37798771 10.1186/s13071-023-05970-3 PMC10552355

[105] Zhou C, Li J, Guo C, Zhou Z, Yang Z, Zhang Y, et al. Comparison of intestinal flora between patients with chronic and advanced *Schistosoma japonicum* infection. Parasit Vectors. 2022;15:413. 36345042 10.1186/s13071-022-05539-6 PMC9640844

[106] Adebayo AS, Survayanshi M, Bhute S, Agunloye AM, Isokpehi RD, Anumudu CI, et al. The microbiome in urogenital schistosomiasis and induced bladder pathologies. PLoS Negl Trop Dis. 2017;11:e0005826. 28793309 10.1371/journal.pntd.0005826 PMC5565189

[107] Cortés A, Martin J, Rosa BA, Stark KA, Clare S, McCarthy C, et al. The gut microbial metabolic capacity of microbiome-humanized vs. wild type rodents reveals a likely dual role of intestinal bacteria in hepato-intestinal schistosomiasis. PLoS Negl Trop Dis. 2022;16:e0010878. 36279280 10.1371/journal.pntd.0010878 PMC9633004

[108] Metabolic reprogramming of hepatocytes by *Schistosoma mansoni* eggs. PubMed. https://pubmed.ncbi.nlm.nih.gov/36590323/. Accessed 21 Nov 2023. 10.1016/j.jhepr.2022.100625 PMC9800334 36590323

